# Insomnia in chronic renal patients on dialysis in Saudi Arabia

**DOI:** 10.1186/1740-3391-8-7

**Published:** 2010-06-14

**Authors:** Hamdan H Al-Jahdali, Haithm A Khogeer, Waleed A Al-Qadhi, Salim Baharoon, Hani Tamim, Fayez F Al-Hejaili, Saeed M Al-Ghamdi, Abdullah A Al-Sayyari

**Affiliations:** 1 Division of Pulmonary/Sleep Disorders Center, King Saud University for Health Sciences. Riyadh- Saudi Arabia; 2 College of Medicine, King Saud University for Health Sciences. Riyadh- Saudi Arabia; 3 Department of Medicine King Saud University for Health Sciences. Riyadh- Saudi Arabia; 4 College of Medicine, King Saud University for Health Sciences, Epidemiology and Biostatistics. Riyadh- Saudi Arabia; 5 Nephrology Division/King Saud University for Health Sciences. Riyadh- Saudi Arabia; 6 Nephrology Division/King Faisal and Research Center Hospital, Jeddah - Saudi Arabia

## Abstract

**Background:**

Studies have shown that insomnia is a common sleep disorder among patients with end-stage renal disease (ESRD). This study aimed to assess the prevalence of insomnia in Saudi patients with ESRD who are on maintenance dialysis.

**Methods:**

This was an observational cross-sectional study carried out over a period of five months in two hemodialysis centers in Saudi Arabia. To assess the prevalence of insomnia, we used the ICSD-2 definition. We also examined the association between insomnia and other sleep disorders, the underlying causes of renal failure, dialysis duration, dialysis shift, and other demographic data.

**Results:**

Out of 227 enrolled patients, insomnia was reported by 60.8%. The mean patient age was 55.7 ± 17.2 years; 53.7% were male and 46.3% were female. Insomnia was significantly associated with female gender, afternoon hemodialysis, Restless Legs Syndrome, high risk for obstructive Sleep Apnea Syndrome and excessive daytime sleepiness (***P-values: ***0.05, 0.01, < 0.0001, < 0.0001, and < 0.0001, respectively). No significant association was found between insomnia and other variables, including BMI, smoking habits, underlying etiology of renal failure, dialysis duration, association with hemoglobin, ferritin, and phosphorus or dialysis adequacy as measured by the Kt/V index.

**Conclusion:**

Insomnia is common in dialysis patients and was significantly associated with other sleep disorders. Greater attention needs to be given to the care of dialysis patients with regard to the diagnosis and management of insomnia and associated sleep disorders.

## Background

End-stage chronic renal disease (ESRD) is a significant problem in Saudi Arabia. In 1986, the prevalence of ESRD was 139 per million people[1]. The number of patients receiving hemodialysis therapy in Saudi Arabia has increased by approximately 10- to 15-fold since 1983, with an estimated annual increase of approximately 8.6% [2,3].

Insomnia is characterized by one or more of the following symptoms: difficulty falling asleep ("sleep onset insomnia"), difficulty staying asleep ("sleep maintenance insomnia"), early awakening or poor sleep quality ("non-restorative sleep") [4,5]. Insomnia is primarily a clinical diagnosis and is most frequently diagnosed using data obtained from patient histories and sleep diaries. The prevalence estimates of insomnia vary because of differences in definition, diagnosis, population characteristics, and research methodologies. Insomnia is a common sleep problem, however, and its prevalence in the general population ranges from 4% to 64% [6,7]. The prevalence of insomnia is substantially greater in dialysis patients and has been reported to range from 45% to 59% [8-10].

Insomnia in uremic patients has greater daytime consequences compared to the general population [11,12]. Elderly patients, those with longer dialysis durations, dialysis shift, and those with high levels of parathyroid hormone (PTH) or diabetes mellitus are at higher risk of insomnia; however, the dialysis type and biochemical parameters are not important determinants of insomnia [10,13]. Other sleep disorders, such as sleep apnea syndrome (SAS) and restless legs syndrome (RLS), are common in dialysis patients and represent the main risk factor for insomnia [12,14]. The prevalence of insomnia due to RLS in dialysis patients ranges from 57% to 83% [10,15,16]. Poor sleep, defined by the Pittsburgh Sleep Quality Index (PSQI)[17], has been reported in 71% of patients on maintenance dialysis [8,9].

Insomnia is associated with a substantial impairment in quality of life (QOL)[18]. It may cause personal distress and adverse social and economic consequences, leading to a number of deleterious effects on behavior, health, sense of well-being, enjoyment of interpersonal relationships and personal safety [18-21]. Severe insomnia can impair daytime functioning and increase the occurrence of accidents and decrease QOL [18,22,23].

A growing number of patients receive dialysis therapy in the Kingdom of Saudi Arabia, and these patients are likely to be affected by insomnia. Many patients may have impairment in daytime function and a reduced QOL. Thus, it is important to know the prevalence and severity of this disorder among dialysis patients.

The aim of the study was to determine the prevalence of insomnia in Saudi patients with ESRD on maintenance dialysis and to assess the association between other sleep disorders, particularly RLS and sleep breathing disorders, and insomnia. We also examined the relationship between insomnia and dialysis duration, underlying medical problems and biochemical parameters.

## Methodology

We conducted an observational cross-sectional study between May 2007 and September 2007 at King Abdulaziz Medical City, King Fahad National Guard Hospital (KAMC-KFNGH), Riyadh, and King Faisal Specialist Hospital and Research Centre (KFHRC), Jeddah. This study was approved by the research and ethics committee at King Abdulaziz Medical City, King Fahad National Guard Hospital (KAMC-KFNGH), Riyadh. We enrolled all stable patients undergoing routine dialysis at both centers. We excluded confused or demented patients or those who refused to participate. Data collection was carried out by personal professional interviews (H.K. or W.Q.) using a structured questionnaire. These questionnaires were adopted from validated international questionnaires and are used routinely at our sleep disorders center. All questionnaires were translated from English to Arabic and back-translated from Arabic to English by a professional medical translator. Moreover, these questionnaires were reviewed by two sleep specialists to confirm the accuracy and clarity of the Arabic translation. Finally, these questionnaires were pre-tested on 30 patients and modified for any ambiguity or vagueness.

The data collected included common demographic characteristics such as age, gender, education level, marital status, employment, and personal habits. Data regarding past medical history, medication, underlying causes of chronic renal failure, dialysis duration and dialysis shift were also collected.

We used the ICSD-2 definition for insomnia, which consists of difficulty in falling asleep, waking up too early, frequent awakening with difficulty in falling asleep again and secondary daytime impairment related to nighttime sleep difficulties [24]. Patients were considered to have insomnia if the symptom frequency was three or more times per week and present for at least one month. Information was also obtained about RLS symptoms using four questions proposed by The International Restless Legs Syndrome Study Group (IRLSSG) for the clinical diagnosis of RLS [25]. Patients were also asked about symptoms of excessive daytime sleepiness using the Epworth Sleeping Scale (ESS), where a score of more than 10 indicates increased sleepiness [26]. Berlin questionnaires were used to assess the risk of Obstructive Sleep Apnea (OSAS) [27,28], and the Pittsburgh Sleep Quality Index (PSQI) was used to assess sleep quality (a score 5 or more indicates poor sleep quality) [17].

Data were summarized as the mean and standard deviation or number and percent, as appropriate. To assess the possible influence of demographic and other variables on the prevalence of insomnia, we used either the unpaired t-test or the Mann-Whitney U-test for non-parametric data, as appropriate. A multivariate logistic regression analysis was used to assess the risk of insomnia while controlling for other parameters, including ESS (score higher than 10 indicates EDS), risk for sleep apnea (using Berlin Questionnaire, high risk vs. low risk), and PSQI (a score higher than 5 indicates poor sleep quality). A p-value of less than 0.05 was considered statistically significant. Data management and analyses were carried out using the Statistical Package for Social Sciences (SPSS), version 13.

## Results

The number of patients included in the study was 227. We found 138 patients who met the ICSD-2 definition of insomnia, yielding a prevalence of 60.8%. Table 1 summarizes the demographic characteristics of the patients included in the study. The mean age was 55.7 ± 17.2 years, and 122 patients (53.7%) were male. The majority of patients (80%) had not completed high school, and 50.7% were employed.

**Table 1:** Demographic characteristics for the study sample, stratified by insomnia status

		All patients N = 227	Insomnia N = 138	No insomnia N = 89	P value
					
**Age**	Mean ± SD	55.7 ± 17.2	55.8 ± 17.7	55.6 ± 16.6	0.92
					
**Gender**	Male	122 (53.7%)	67 (48.6%)	55 (61.8%)	0.05
	Female	105 (46.3%)	71 (51.4%)	34 (38.2%)	
					
**Marital Status**	Married	25 (11.0%)	16 (11.6%)	9 (10.1%)	0.15
	Single	155 (68.3%)	88 (63.8%)	67 (75.3%)	
	Divorced/Widowed	47 (20.7%)	34 (24.6%)	13 (14.6%)	
					
**Education level**	Illiterate	100 (44.1%)	63 (45.7%)	37 (41.6%)	0.83
	Elementary	46 (20.3%)	29 (21.0%)	17 (19.1%)	
	Preparatory	35 (15.4%)	21 (15.2%)	14 (15.7%)	
	High school	22 (9.7%)	13 (9.4%)	9 (10.1%)	
	University	24 (10.6%)	12 (8.7%)	12 (13.5%)	
					
**Occupation**	Employed	115 (50.7%)	65 (47.1%)	50 (56.2%)	0.51
	Unemployed	112 (49.3%)	73 (52.9%)	39 (43.8%)	

The mean duration of time on dialysis was 40.4 ± 37.8 months. The most common cause of renal failure was diabetes mellitus (DM) (52%).

There was no significant association between insomnia and age, BMI, underlying medical problems, behavioral factors such as smoking habits, coffee intake or dialysis type. In addition, no association was found between insomnia and sleep adequacy, as measured by the Kt/v index and other biochemical parameters, including hemoglobin, ferritin, phosphorus and calcium levels. However, insomnia was significantly more frequent in females and patients with the afternoon dialysis shift (Tables 1 and 2).

**Table 2:** Association between insomnia status and other characteristics (shift and type of dialysis, lifestyle habits, and underlying medical problems)

Characteristics		Insomnia N = 138	No insomnia N = 89	OR	CI	P value
						
**Dialysis**						
Shift	Morning	60 (54.1%)	29 (37.7%)	Ref	Ref	-
	Afternoon	30 (27.0%)	35 (45.5%)	0.41	0.21 - 0.80	0.009
	Evening	21 (18.9%)	13 (16.9%)	0.78	0.34 - 1.78	0.56
						
Type	Hemodialysis	111 (80.4%)	77 (86.5%)	Ref	Ref	-
	Peritoneal	27 (19.6%)	12 (13.5%)	1.56	0.75 - 3.27	0.24
						
Duration of dialysis	≤ 12 months	50 (36.2%)	27 (30.3%)	Ref	Ref	-
	13-48 months	48 (34.8%)	38 (42.7%)	0.68	0.36 - 1.28	0.24
	49-84 months	21 (15.2%)	18 (20.2%)	0.63	0.29 - 1.38	0.25
	> 84	19 (13.8%)	6 (6.7%)	1.71	0.61 - 4.79	0.31
						
**Lifestyle characteristics**						
BMI categories	Normal	60 (43.5%)	39 (43.8%)	Ref	Ref	-
	Overweight	46 (33.3%)	26(29.2%)	1.15	0.61 - 2.15	0.66
	Obese	32 (23.2%)	24 (27.0%)	0.87	0.45 - 1.69	0.67
						
Smoking	Smoker	28 (20.3%)	24 (27.0%)	Ref	Ref	-
	Ex/Nonsmoker	110 (79.7%)	65 (73.%)	0.69	0.37 - 1.29	0.24
						
Coffee intake	Yes	102 (73.9%)	70 (78.7%)	Ref	Ref	-
	No	36 (26.1%)	19 (21.3%)	0.77	0.41 - 1.45	0.42
						
**Medical conditions**						
DM	Yes	73 (52.9%)	46 (51.7%)	Ref	Ref	-
	No	65 (47.1%)	43 (48.3%)	1.05	0.62 - 1.79	0.86
						
Hypertension	Yes	117 (84.8%)	73 (82.0%)	Ref	Ref	-
	No	21 (15.2%)	16 (18.0%)	1.22	0.60 - 2.49	0.58
						
Erythropoietin & iron supply	Yes	135 (97.8%)	84 (94.4%)	Ref	Ref	-
	No	3 (2.2%)	5 (5.6%)	2.68	0.62 - 11.5	0.19
						
**Biochemical parameters**						
KTV (Hemodialysis)	≥1.2	89 (89.9%)	65 (89.0%)	Ref	Ref	-
	< 1.2	10 (10.1%)	8 (11.0%)	0.91	(0.34-2.44)	0.86
						
KTV (Peritoneal dialysis)	≥1.7	15 (75.0%)	7 (77.8%)	Ref	Ref	-
	< 1.7	5 (25.0%)	2 (22.2%)	1.17	(0.18-7.56)	0.87
						
Albumin	Normal	118 (87.4%)	74 (85.1%)	Ref	Ref	-
	Low	17 (12.6%)	13 (14.9%)	0.82	(0.38-1.79)	0.62
						
Hgb	Normal	59 (44.7%)	34 (40.5%)	Ref	Ref	-
	Low	73 (55.3%)	50 (59.5%)	0.84	(0.48-1.47)	0.54
						
	2.10-2.55 (Normal)	90 (66.7%)	63 (74.1%)	Ref	Ref	-
Calcium	< 2.10	33 (24.4%)	20 (23.5%)	1.16	(0.61-2.20)	0.66
	> 2.55	12 (8.9%)	2 (2.4%)	4.20	(0.91-19.42)	0.05
						
	Normal	66 (50.0%)	40 (48.2%)	Ref	Ref	-
Phosphorus	Low	8 (6.1%)	7 (8.4%)	0.69	(0.23-2.06)	0.51
	High	58 (43.9%)	36 (43.4%)	0.98	(0.55-1.73)	0.94
						
Ferritin						
	Normal	42 (33.6%)	29 (35.8%)	Ref	Ref	-
	Low	0 (0.0%)	2 (2.5%)	-	-	-
	High	83 (66.4%)	50 (60.2%)	1.15	(0.64-2.07)	0.65

As shown in Table 3, the multivariate analysis showed a significant independent association between insomnia and RLS, OSAS and EDS (***P-values ***= 0.01, 0.02, and 0.03, respectively). All patients with insomnia had poor sleep quality, as measured by the Pittsburgh Sleep Quality Index (PSQI). We considered insomnia symptoms to be particularly significant if they were present for more than one month. The most common insomnia symptoms were awakening from sleep too early, reported by 58.6% of patients, and frequent awakening with difficulty in falling asleep again, reported by 57.7% of patients. A summary of patient responses to questions about insomnia and duration of symptoms are shown in Table 4.

**Table 3:** Association between insomnia and other sleep disturbances

Other sleep disorders		Insomnia N = 138	No insomnia N = 89	OR	CI	P value
						
RLS	Positive	86 (62.3%)	28 (31.5%)	Ref	Ref	-
	Negative	52 (37.7%)	61 (68.5%)	2.18	1.18 - 4.06	0.01
						
PSQI	Good Sleep	0 (0.0%)	3 (3.4%)	Ref	Ref	-
	Bad Sleep	138 (100.0%)	86 (96.6%)	NA	NA	NA
						
Berlin	Low risk	22 (16.2%)	36 (43.4%)	Ref	Ref	-
	High risk	114 (83.8%)	47 (56.6%)	2.10	1.15 - 3.81	0.02
						
ESS	Positive	78 (56.5%)	22 (24.7%)	Ref	Ref	-
	Negative	60 (43.5%)	67 (75.3%)	2.13	1.09 - 4.15	0.03

**Table 4:** Patient responses to insomnia questions

	Total = 227	< 1 month	> 1 month*
1- Do you often have difficulty falling asleep?	121 (53.3%)	5 (2.2. %)	116 (51.1%)
2- Do you have frequent awakening with difficulty falling asleep again?	131 (57.7%)	5 (2.2%)	126 (55.5%)
3- Do you wake up too early?	133 (58.6%)	4 (1.7%)	129 (56.8%)
4- Do you often feel tired when you awaken in the morning due to sleep loss?	147 (64.8%)	6 (2.6%)	141 (62.1%)
5- Does sleep loss affect your mood during the day, making you feel tense, irritable or depressed?	107 (47.1%)	2 (0.88%)	105 (46.2%)

*Some patients had more than one answer

## Discussion

To the best of our knowledge, no similar study has investigated the prevalence of insomnia in patients on maintenance dialysis in Saudi Arabia. The prevalence of insomnia in dialysis patients has been reported to range between 45% and 59% [8-11]. Furthermore, clinically significant insomnia (with daytime consequences) has been reported to be higher in dialysis patients than normal population [4]. This study suggests that insomnia in dialysis patients may have a greater daytime consequence than in the general population. The overall prevalence of clinically significant insomnia in our study was 60.8%, which is higher than the rate reported by other studies. This difference is probably due to the high prevalence of RLS in our study (62%) and to the longer dialysis duration (more than 12 months in 64% of patients). Sabbatini et al. [10] reported an association between longer dialysis duration and insomnia. In his systemic review of symptoms in ESRD, Murtagh et al. [29] demonstrated that fatigue/tiredness was the most common complaint (reported in 70% of cases). Several factors are associated with the fatigue/tiredness experienced in ESRD, including gender, age, diabetes, prescribed medications and their side effects, nutritional deficiencies, physiological alterations (particularly abnormal urea and anemia), and psychological factors such as depression and sleep disorders [10,30]. Diabetes as an underlying cause of chronic renal failure and anemia has been associated with symptoms of fatigue and tiredness [31]. In our study, no association was found between insomnia, diabetes, and anemia. We did not assess our patients for depression; therefore, depression as a cause of fatigue, tiredness, and sleep loss could not be ruled out.

A study by Sabbatini et al. [10] reported a higher prevalence of insomnia in females and older patients., We found no association between insomnia and age, however, in our study. Similar to other studies, females were more strongly affected than males [10], a result that was marginally significant (*P-value *= 0.05).

Sabbatini et al. [10] reported a higher rate of insomnia among patients on the morning dialysis shift; however, in our study, those with the afternoon dialysis had a higher rate of insomnia than any other shift. This difference in the effect of dialysis shift may relate to sleep schedules in different societies. In Saudi Arabia, it is common for many people to go to sleep late at night and take afternoon naps, particularly unemployed individuals (in our study, only 50.7% of the studied patients were employed). Patients who have afternoon dialysis shifts may be deprived of afternoon naps, which may lead to feelings of sleepiness and general fatigue/tiredness.

We observed a significant association between insomnia and the presence of RLS, high risk for OSAS and excessive daytime sleepiness (***P-value ***< 0.0001). The prevalence of RLS was 50% in all studied patients and 62% in patients with insomnia. This finding is similar to other studies, where RLS was reported in 45-83% of insomnia patients [10,15,16].

The main strengths of this study are the large number of recruited patients and the comprehensive personal interviews conducted by trained professionals. Another strength is that, to the best of our knowledge, this is the first study to address this issue in Saudi patients. An important limitation of this study is the absence of certain vital information, including depression and other causes of daytime fatigue, because sleep diaries were not utilized. Another limitation is using translated questionnaires which are not validated for our population; in addition, we had no group from the general population to serve as a control group. Despite these limitations, this study confirms the high prevalence of clinically significant insomnia in Saudi patients undergoing dialysis.

## Conclusions/Recommendations

Our study clearly demonstrates that the prevalence of clinically significant insomnia among Saudi dialysis patients is high (60.8%), and most cases were associated with other sleeping disorders, such as RLS and OSAS. Identifying and treating other underlying sleeping disorders may improve insomnia and therefore patient quality of life and also decrease the prevalence of complications related to poor sleep quality.

## Competing interests

The authors declare that they have no competing interests.

## Authors' contributions

HJ: Is the principle investigator, designed the study, and wrote the questionnaires and the initial manuscript

HK and WQ: Interviewed the patients

SB: Participated in the study design and writing the manuscript

HT: Reviewed the statistics and the manuscript

FH: Coordinated and facilitated interviews with the dialysis patients and provided all laboratory data at Riyadh

SG: Coordinated and facilitated interviews with the dialysis patients and provided all laboratory data at Jeddah

AS. Critically reviewed the manuscript and supervised the students

**All authors read and approved the final manuscript**.
